# Seasonal Foraging Ecology of Non-Migratory Cougars in a System with Migrating Prey

**DOI:** 10.1371/journal.pone.0083375

**Published:** 2013-12-12

**Authors:** L. Mark Elbroch, Patrick E. Lendrum, Jesse Newby, Howard Quigley, Derek Craighead

**Affiliations:** 1 Panthera, New York, New York, United States of America; 2 Craighead Beringia South, Kelly, Wyoming, United States of America; Université de Sherbrooke, Canada

## Abstract

We tested for seasonal differences in cougar (*Puma concolor*) foraging behaviors in the Southern Yellowstone Ecosystem, a multi-prey system in which ungulate prey migrate, and cougars do not. We recorded 411 winter prey and 239 summer prey killed by 28 female and 10 male cougars, and an additional 37 prey items by unmarked cougars. Deer composed 42.4% of summer cougar diets but only 7.2% of winter diets. Males and females, however, selected different proportions of different prey; male cougars selected more elk (*Cervus elaphus*) and moose (*Alces alces*) than females, while females killed greater proportions of bighorn sheep (*Ovis canadensis*), pronghorn (*Antilocapra americana*), mule deer (*Odocoileus hemionus*) and small prey than males. Kill rates did not vary by season or between males and females. In winter, cougars were more likely to kill prey on the landscape as: 1) elevation decreased, 2) distance to edge habitat decreased, 3) distance to large bodies of water decreased, and 4) steepness increased, whereas in summer, cougars were more likely to kill in areas as: 1) elevation decreased, 2) distance to edge habitat decreased, and 3) distance from large bodies of water increased. Our work highlighted that seasonal prey selection exhibited by stationary carnivores in systems with migratory prey is not only driven by changing prey vulnerability, but also by changing prey abundances. Elk and deer migrations may also be sustaining stationary cougar populations and creating apparent competition scenarios that result in higher predation rates on migratory bighorn sheep in winter and pronghorn in summer. Nevertheless, cougar predation on rare ungulates also appeared to be influenced by individual prey selection.

## Introduction

Ungulate migrations, driven by the seasonal availability of forage, result in large-scale redistributions of resources for carnivores, and in response, carnivores exhibit variable foraging behaviors in systems with migrating prey [1]. Carnivore responses to migratory prey, in turn, influence predator-prey dynamics, including predator functional responses and apparent competition influencing rare prey during seasonal overlap with more abundant prey (e.g., cougar, *Puma concolor*, and bighorn sheep, *Ovis canadensis*, [2]). On the one hand, migrating prey change prey availability for non-migratory predators, and on the other, predators that select different habitats in different seasons in response to migrating prey, also change what prey are available to them. Some populations of African lions (*Panthera leo*) [3], cougars [4], and wolves (*Canis lupus*) [5] migrate with their primary prey. Other populations remain in place, limited in their movements while attending young [6]. Regardless of whether predators follow prey migrations, individual animals and groups of animals within each species exhibit variation in their response to migrating prey. For example, some wolf packs remain in place and hunt secondary prey while their primary prey migrates away, others remain in place yet travel great distances to hunt their primary prey, and others follow with their migrating prey [7]. 

Previous research has shown that some terrestrial carnivores exhibit seasonal variation in prey selection and kill rates dependent upon seasonal variation in prey vulnerability [8,9,10]. Thus, predator-prey modeling based upon sampling in a single season may lead to inaccurate conclusions [10]. Winter prey selection in North America and dry-season prey selection in Africa is driven by the increased availability of animals of poorer health [8,10], and seasons in which ungulates give birth provide a pulse of vulnerable, smaller prey [11]. Whereas previous research has focused upon prey availability in terms of prey vulnerability, here we assess predator foraging ecology as affected by prey availability driven by ungulate migrations.

Cougars are a solitary felid and occupy the largest geographic range of any terrestrial mammal in the western hemisphere, exhibiting plasticity in habitat use and prey selection [12]. Cougar populations are primarily non-migratory and hunt non-migratory prey [12]. Nevertheless, where mule deer (*Odocoileus hemionus*) exhibit seasonal migrations, some cougars follow them, exhibiting seasonal ranges themselves; other cougars remain in winter deer range through the summer, where deer persist, but at lesser numbers [4,13]. Cougars also exhibit numerous seasonal foraging strategies, including seasonal prey selection [11], seasonal kill rates [11], and seasonal habitat use [4,13]. Whereas previous research on other predators have emphasized that predators select different prey in different seasons due to variation in prey vulnerability [8,10], cougars as a species do not always select disadvantaged prey exhibiting physical vulnerability. Instead, cougars select prey opportunistically (i.e., of any health) in areas where structural complexity (e.g., slope, trees, boulders) provide them an advantage [14,15,16]. Therefore, it is logical to hypothesize that cougar seasonal prey selection and kill rates may be driven by prey availability in terms of actual prey numbers, as well as prey vulnerability.

Our goals were to test for differences in a suite of seasonal cougar foraging behaviors, including prey selection, kill rates, and habitat use, in the Southern Yellowstone Ecosystem (SYE), a multi-prey system in which many ungulate prey migrate, and cougars remain in place. Our study system included numerous potential prey for cougars, including abundant elk (*Cervus elaphus*) and numerous mule deer, and smaller populations of bighorn sheep, moose (*Alces alces*) and pronghorn (*Antilocapra americana*). All of these species, except moose, exhibit seasonal migrations within the study area [17,18,19]. Elk migration is in part due to historic and ongoing food subsidies provided them on and adjacent the National Elk Refuge in winter. Due to their low and declining numbers, moose, pronghorn, and bighorn sheep are of critical conservation concern, and elk and mule deer, too, are exhibiting declines [20,21]. Thus a better understanding of patterns of predation on ungulates in the SYE, including the influence of ungulate migrations on seasonal cougar prey selection, holds immediate conservation value.

We defined prey specialization as killing a prey species in greater numbers than any other prey [22], and we hypothesized that unlike elsewhere in their range, cougars would specialize on deer in summer but elk in winter. We also hypothesized that cougar kill rates would be higher in summer than winter because of selecting for smaller, younger ungulates with less energetic value than adult animals [11]. Further, we hypothesized that cougar predation on less abundant bighorn sheep would be highest in winter and predation on pronghorn would be highest in summer, when these species migrated into the range of stationary, resident cougars monitored as part of our study. We expected predation on moose to occur equally across seasons because they did not migrate in and out of the study area. Last, we hypothesized that cougars would utilize different habitats for hunting in summer versus winter, because of changes in prey assemblage, distributions, and availability. 

## Methods

### Ethics statement

Our capture protocols for cougars, a species which is neither threatened or endangered, followed those outlined in Quigley [23], adhered to the guidelines outlined by the American Society of Mammalogists [24], and were approved by the Jackson Institutional Animal Care and Use Committee (Protocol 027-10EGDBS-060210). Every effort to ameliorate suffering of cougar subjects was made, and no cougars were ever killed/sacrificed as part of research methods. Our research was carried out on the Bridger-Teton National Forest (United States Forest Service, USFS Authorization ID JAC760804), Grand Teton National Park (NPS Permit GRTE-2012-SCI-0067), and National Elk Refuge (USFW permit NER12),with permission to handle cougars granted by the Wyoming Game and Fish Department (ID 297).

### Study area

**Figure 1:**
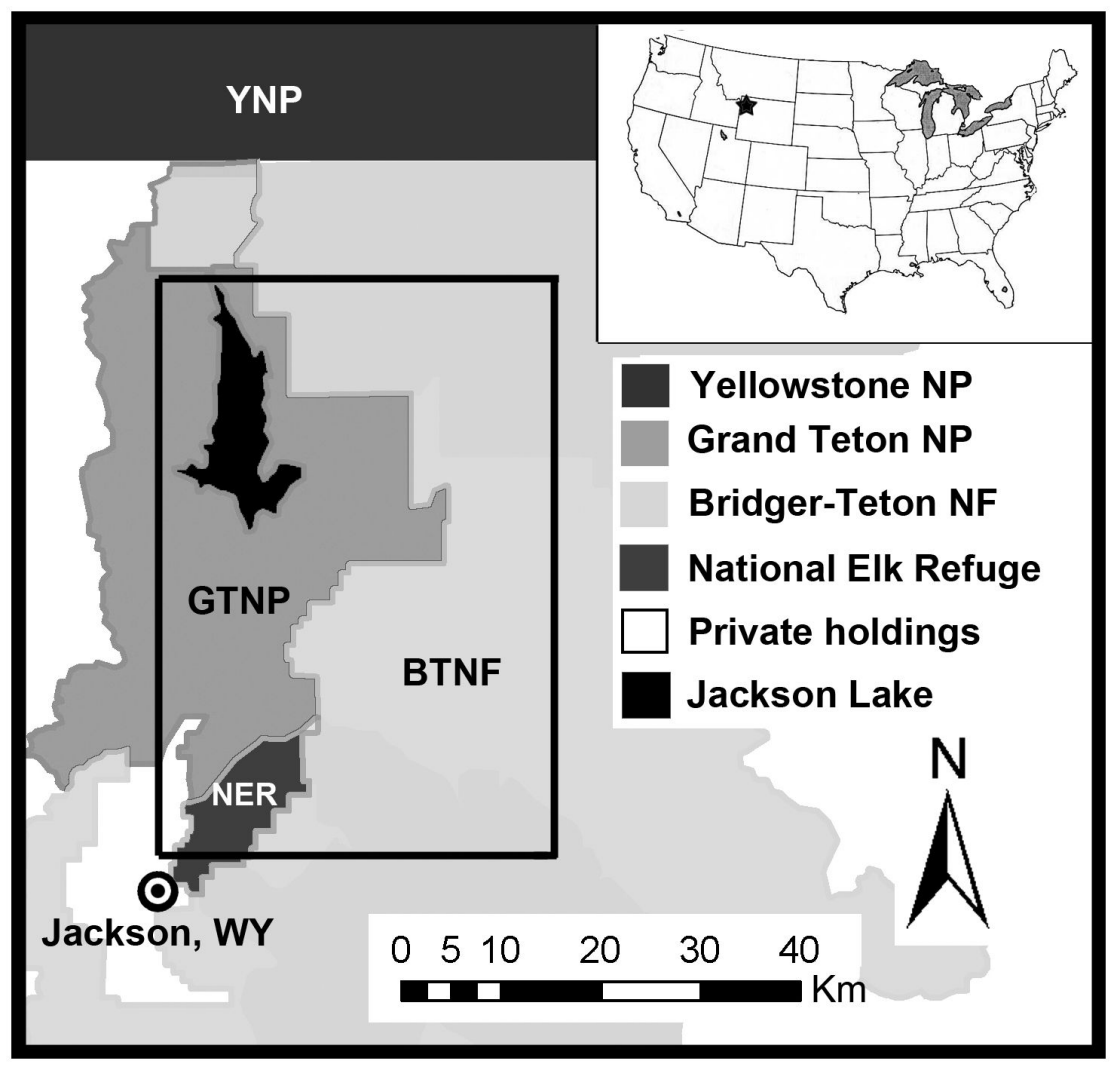
Location of the study area in northwest Wyoming, USA. Location of the study area in northwest Wyoming, USA, and a close up of land ownership within the area of focus. The smaller rectangle delineated by a black line was the area in which we focused capture efforts and our interaction study using marked individuals.

Our study area encompassed approximately 2,300 km^2^ of the Southern Yellowstone Ecosystem (SYE), inclusive of Grand Teton National Park (United States Park Service), the National Elk Refuge (United States Fish and Wildlife Service), and the Bridger-Teton National Forest (United States Forest Service) north of the town of Jackson, Wyoming (Figure 1). Elevations in the study area ranged from 1,800 m in the valleys to > 3,600 m in the mountains. Plant communities included cottonwood (*Populus angustifolia*) riparian zones interspersed with sagebrush (*Artemisia spp.*) uplands at lower elevations. At intermediate elevations, aspen (*P. tremuloides*), Douglas-fir (*Pseudotsuga menziesii*), and lodgepole pine (*Pinus contorta*) were the dominant species. Spruce (*Picea engelmannii*) and fir (*Abies lasiocarpa*) were the primary tree species at the higher elevations [25]. The area was characterized by short, cool summers and long winters with frequent snowstorms. Precipitation occurred mostly as snow, and mean maximum snow depths ranged from 100 cm at lower elevations to > 245 cm at intermediate and higher elevations (2,000 m +). 

The study area supported a diverse community of large mammals. Carnivores included wolves, black bears (*Ursus americanus*), grizzly bears (*U. arctos*), coyotes (*Canis latrans*), and red foxes (*Vulpes vulpes*). Ungulates included elk, mule deer, moose, bison (*Bison bison*), pronghorn, bighorn sheep, and a very small number of white-tailed deer (*Odocoileus virginianus*). Deer, elk, bighorn sheep, and pronghorn exhibited seasonal migrations [7,17,18,19]. 

### Cougar capture and collar programming

Beginning in 2001, we captured cougars with permission from the Wyoming Game and Fish Department during winters, from late-November through March of the following year on the Bridger-Teton National Forest, when determining the presence of a cougar was facilitated by snow. We used trailing hounds to force cougars to retreat to a tree or rocky outcrop where we could safely approach them. Cougars were immobilized with ketamine (4.0 mg/kg) and medetomidine (0.07 mg/kg), and then their temperature, heart rate, and respiration were monitored at 5 minute intervals while they were processed, sampled, and fitted with either a VHF (Telonics, Mesa, AZ) or GPS collar (Telonics, Mesa, AZ; Televilt, Bandygatan, Sweden; Vectronics, Berlin, Germany). Once an animal was completely processed, the effects of the capture drugs were reversed with Atipamezole (0.375 mg/kg), and cougars departed the capture sites on their own. 

### Determining cougar prey selection

We conducted site searches of areas on the Bridger Teton National Forest, Grand Teton National Park, and National Elk Refuge, where triangulation of cougars wearing VHF collars revealed that they had not moved for 24+ hrs, or spatially aggregated GPS points, called GPS clusters [26], indicated a cougar had remained in place for 8+ hours. Prey remains, including hair, skin, rumen (stomach), and bone fragments, were used to identify prey species. We determined sex of ungulate prey using external morphology, and the relative age of prey using tooth eruption sequences and wear in the lower mandible [27] as follows: < 1 yr of age (young of the year), 1-3 yrs (subadult), > 3yrs (adult). 

### Testing for differences in prey selection between males/females and summer/winter

Following well-established elk migration dates in the study area, we defined winter as Dec 1^st^ of one year through May 31^st^ of the next year, and summer as June 1^st^ through November 30^th^ of the same year. To account for variable number of prey killed by individual cougars, we quantified each individual cougar’s prey selection as the proportions of prey killed by each cougar, before conducting any population level analyses. We employed two-proportion z-tests to account for variable numbers of kills between males and females, and winter and summer, to test whether female and male cougars selected different proportions of different prey, and then whether males and females selected different proportions of prey in summer versus winter.

### Estimating kill rates and testing for seasonal variation

We estimated seasonal kill rates for cougars wearing GPS collars with models that differentiated between GPS clusters with a high probability of being a kill versus GPS clusters with a low probability of being a kill. GPS collars were programmed to acquire location data at variable intervals, ranging from every 3 to 10 hour intervals, with the majority of collars being programmed to acquire 6-8 locations each day. To identify clusters, GPS data were analyzed with a Python script (Python Software Foundation Hampton, NH) developed by Knopff et al. [28] to identify 2 or more locations within 100 meters and 48 hrs of each other. We did not include cougars wearing VHF collars in our kill rate calculations.

 First, we developed competing models to test 4 spatial and 7 temporal attributes of clusters potentially predictive of kill sites (Table 1) [26,29,30]. We tested these models against our database of a subset of GPS clusters we visited in the field, and for which we determined whether or not a kill was present. Additional sites where the presence of a kill could be confirmed were provided by visits based on VHF radio telemetry for clusters later identified with GPS data stored on collars. For cluster analysis, site searches conducted ≥ 4 months from the beginning date of cluster formation and site searches of questionable reliability (eg. when snow fall may have obscured evidence) were omitted. 

**Table 1:** Spatial and temporal attributes of clusters examined as potential predictors for cougar kills.

**Variable name**	**Variable description**
Duration (hours)	Number of hours in cluster
Day-period	Number of 24 hour periods in cluster
Number of nights	Number of separate nights (19:00 – 7:00hrs) in cluster
Days>1	True/False identifying clusters including more than one 24 hour period
Nights>1	True/False identifying clusters including more than one night (19:00 – 7:00hrs)
Night 1+	True/False identifying clusters including at least one night (19:00 – 7:00hrs)
Night hours	Number of hours in cluster occurring between 19:00 – 7:00hrs
Fidelity	Proportion of locations at cluster site vs. away over time frame of cluster
Cluster radius	Radius of cluster in meters
Average distance from centroid	Average distance in meters between locations and the center of the cluster
Nearest cluster	Distance in meters to closest consecutive cluster

We employed logistic regression to assess the ability of univariate and multivariate models to predict at which clusters we would likely find a cougar kill. We employed residual plots of univariate models to assess assumptions of linearity and normality and transformed data accordingly. We also excluded models in which variables exhibited collinearity ≥ 0.7. We applied backwards and forwards model selection to select the best supported model, based on Akaike’s Information Criterion, and obtain parameter estimates. We did not use weighted parameter estimates for predictor variables because of the large number of highly correlated variables. We assessed the fit of the top model from receiver operative characteristic (ROC) curves [31]. 

We employed sensitivity and specificity curves to assess the efficiency of models in predicting kill sites and determining the optimum probability cut-point for clusters to identify kills [31]. High sensitivity may effectively classify clusters with kills successfully (reduce false-negatives), but is likely to attribute kills to clusters without them (increase false-positives). In contrast, high specificity may result in more false negatives, but few false positives.

We applied our final model to GPS cluster data for all individuals that wore GPS collars to predict which clusters were likely to contain kills as follows: Pr(kill) = exp^(β0 + β1*X1 + β2*X2 + βn*Xn^)/1 + exp^(β0 + β1*X1 + β2*X2 + βn*Xn)^. Where β_0_ is the intercept and β_n_ are coefficients for predictor variables X_n_ [32]. We applied this model to identify clusters that likely contained kills in the entire GPS location data from all cats. We used these identified clusters in the full GPS dataset to estimate seasonal kill rates for each individual cougar. We conducted a test of whether there were differences in kill rates due to season or cougar sex with a 2-way analysis of variance, where individual cougar was included as a random effect.

Due to the low success rate of the final models in classifying clusters, we examined an alternative model for identifying kills. This model was based on previous research by Anderson and Lindzey [26] and Ruth et al. [30], which employed the number of nights a cougar visited a cluster as an efficient means to distinguish between kill and non-kill sites. In this approach, kills were assigned only to clusters that spanned more than one night. We reported kill rates determined using both the “best” and night>1 models, because the benefit of the night>1 model’s ability to positively detect kills at sites may outweigh the cost of over attributing kills to clusters where none were found. As this method likely missed small prey that could be consumed in shorter time frames, we defined these kill rates as ungulate-only kill rates (which may have additionally underestimated the number of fawns, calves and lambs killed by cougars).

### Testing for seasonal cougar space use

We determined seasonal fixed-kernel home ranges for marked, adult cougars resident in the study area from 2001 to 2011 using ArcGIS 10 and the calculation of kernel density estimators (KDE) [33,34] and isopleths in the Geospatial Modeling Environment (GME) [35]. We examined home ranges at 90% KDE. Annual home ranges were calculated for cougars for which we had gathered a minimum of 20 independent locations for each season across at least 10 months in a given year. Cougars with VHF collars were triangulated on the ground and from fixed-wing aircraft. We determined the smoothing factor (h) for VHF data with least squares cross validation (LSCV) [33], and for GPS data, using the Plug-in method [36] in the GME. To test whether cougars remained stationary through the seasons, we quantified their seasonal home range overlap. We defined “stationary” as a mean overlap of 50% or more between summer and winter ranges.

We also quantified the seasonal overlap between the marked cougar population and the seasonal ranges for pronghorn, bighorn sheep, mule deer and elk. We employed the 90% kernel density of all cougar data for summer and winter home ranges, and seasonal ungulate distributions determined by Wyoming Game and Fish Department surveys (wgfd.wyo.gov/web2011/wildlife-1000819.aspx).

### Testing for differences in seasonal cougar hunting areas

We identified 7 landscape variables as potentially important predictors of cougar kill sites: elevation (m), slope (%), aspect (transformed into categories of North, East, South, West), terrain ruggedness (vector ruggedness measure; VRM), vegetation type, distance to water, and distance to edge habitat. We estimated elevation using a digital-elevation model (DEM) at a resolution of 30 m (http://datagateway.nrcs.usda.gov/). We then used ArcGIS 10.0 Spatial Analyst Tools to derive values of slope and aspect from the DEM. In addition, we also derived a vector ruggedness measure (VRM) from the DEM following Sappington et al. [37]. A Gap Analysis Program (GAP) land cover (gapanalysis.usgs.gov/gaplandcover) was used, at a resolution of 30 m, which included 87 cover classes, which we reclassified into 5 cover based on similarity of land cover types: (1) open meadows or crop lands, (2) barren habitats and open water bodies, (3) shrub-steppe, (4) forested, (5) and riparian zones. Water sources included streams and rivers obtained from hydrologic units (http://datagateway.nrcs.usda.gov/). We converted all forested lands from the GAP into a polygon layer in ArcGIS to be used as an indication of cover. We then created edge layers as the perimeter of each forested section. 

Prior to modeling, we used a correlation matrix to evaluate collinearity (|*r*| > 0.7) among predictor variables. No predictor variables were correlated (|*r*| < 0.50) and therefore, all variables remained in the modeling process. We then modeled all possible combinations of the 7 predictor variables. For the categorical variable of aspect, we used southerly aspect as a reference category because southerly aspects are commonly used by prey species [38].

To estimate resource-selection functions for each season, we employed conditional logistic regression [39,40,41] to compare kill sites with random locations, located 2 km from the kill site along each cardinal direction. Each cougar was considered a stratified variable to control for variation among individuals (i.e., individuals were sampling units), and the logistic model for each study area was made condition upon that variable [42,43]. We calculated Akaike’s Information Criterion adjusted for small sample size (*AICc*), *ΔAICc*, and Akaike weights (*w*_*i*_) for each model [44]. We considered models with *ΔAICc* values > 2.0 to measurably differ in information content and then used model-averaged parameter estimates and unconditional standard errors (SE) to assess the influence of each predictor variable on resource selection from the top models [44]. To evaluate predictive strength of the resource-selection functions for kill sites for each season, we used *k*-fold cross validation [45]. We portioned kill sites into five equal sets, and models were fit to 80% partition of the data, while the remaining 20% of the data were used as test data [46,47]. We used RSF scores to rank the observed location of each stratum against the test data. We then regressed the number of locations from the observed dataset in each bin against the median RSF value of the test data, and recorded the coefficient of determination (*r* ^*2*^). Additionally, we calculated a Spearman’s rank correlation (*r*_*s*_) as an additional metric of predictive strength. Values with a high *r* ^*2*^ and *r*_*s*_ were indicative of models with high predictive strength [45]. 

Based on our seasonal RSF results, and with respect to a reference vector, defined as the set of mean values for each variable within the domain of availability, we then calculated the relative probability of a cougar killing prey across the landscape in ArcGIS 10 [46,47]. We converted parameter estimates to odds ratios by exponentiation for simplicity of interpretation. Therefore, if the 95% confidence interval around an odds ratio contained 1, then that variable was considered not significant [43,46]. The resulting odds ratio expression for a given landscape location was then calculated using the spatial distribution of cougar kills to generate a probability surface that served as a template to identify landscape heterogeneity [46,47]. Cells with a higher value indicated a higher relative probability of kill occurrence.

## Results

### Seasonal prey indices and prey selection

Between January, 2001, and October 1, 2012, we recorded 411 winter prey and 239 summer prey killed by 28 female and 10 male cougars, and an additional 37 prey items recorded for unmarked cougars (30 winter, 7 summer). Of these, only 29 prey were not ungulates, and so we lumped them together under “small prey” in analyses. Small prey included 8 North American porcupines (*Erethizon dorsatum*), 6 snowshoe hares (*Lepus americanus*), 3 grouse (*Bonasa* spp.), 3 Northern raccoons (*Procyon lotor*), 2 red squirrel (*Tamiasciurus hudsonicus*), 1 red fox, 1 yellow-bellied marmot (*Marmota flaviventris*), 1 great-horned owl (*Bubo virginianus*), 1 American marten (*Martes americanus*), 1 red-naped sapsucker (*Sphyrapicus nuchalis*), 1 Canada goose (*Branta canadensis*), and 1 cougar.

Mule deer composed 42.4% of summer cougar diets but only 7.2% of winter diets. Elk composed 38.3% of summer cougar diets but 74.4% of winter diets. Males and females, however, selected different proportions of different prey (Table 2); male cougars selected more elk and moose than females, while females killed greater proportions of bighorn sheep, pronghorn, mule deer and small prey than males (Table 3). Seven of 28 females killed bighorn sheep and 3 of 28 females killed pronghorn. Five of 28 females and 4 of 10 males killed moose.

**Table 2:** A comparison of proportions (%) of different prey killed by male and female cougars, and the results of the two-proportions z-test.

	Female	Male	z	P (2-tailed)
Bighorn	12.5	0.0	5.45	<0.001*
Deer	26.6	17.3	5.15	<0.001*
Elk	28.0	42.7	-0.229	0.819
Moose	8.0	36.7	-6.789	<0.001*
Pronghorn	7.9	0.0	2.332	0.0197*
Small prey	16.0	3.4	2.467	0.014*

Significant results are marked with an asterisk (*).

**Table 3:** A comparison of proportions (%) of different prey killed by female and male cougars in summer and winter, and the results of the two-proportions z-test.

Females				
	Summer (*n*=189)	Winter (*n*=329)	z	P (2-tailed)
Bighorn	2.7	8.3	-2.904	0.004*
Deer	43.5	10.2	8.376	<0.001*
Elk	41.6	75.0	-7.747	<0.001*
Moose	0.5	2.1	-1.694	0.09
Pronghorn	2.2	0.1	1.942	0.052*
All small	9.6	4.3	2.191	0.028*
Males				
	Summer (n=50)	Winter (n=82)	z	P (2-tailed)
Deer	17.3	1.3	2.913	0.004*
Elk	58.6	87.6	-3.690	<0.001*
Moose	16.7	10.6	0.9721	0.331
All small	7.5	0.5	1.840	0.066

Significant results are marked with an asterisk (*).

### Cougar kill rates

From 2005 to 2012, we collected location data adequate to identify clusters from 17 cougars wearing GPS collars. During this same time period, we visited 309 clusters from 14 individuals (average clusters visited/cat = 22; range 1-81 clusters/cat) to search for prey remains. Sites were visited between 0 – 98 days after initial cluster formation (average = 8 days). We found kills at 269 clusters and classified 40 clusters as non-kills, and with these data, we tested our kill rate models.

The final logistic regression model for determining if a cluster was likely a kill included the cluster spanning more than one night (*z* = 3.042; P = 0.002), the square root of the number of hours at night in a cluster (*z* = 2.084; P = 0.037), fidelity (*z* = 2.205; P = 0.027), and distance to nearest consecutive cluster (*z* = 2.284; P = 0.022). The probability of finding a kill was positively associated with all 4 variables included in the final model. Model fit was moderate for the final model (AUC = 0.827). The probability cut-off of 0.887 maximized both sensitivity and selectivity in discriminating between clusters with and without kills, however, this cut-off resulted in only a 49.7% success of correctly classifying our known kill and non-kill clusters, identified with field investigations. 

The variable “night>1” was found to be the strongest predictor of a cluster being a kill in both the multivariate models and among all univariate models (R^2^ for night>1 = 0.148; all other independent variables R^2^ < 0.057). The night>1 model was found to successfully identifying clusters as having kills 86% of instances, but attributed kills to 40% of sites at which we did not find prey remains. This resulted in a 46.0% classification success for the night>1 model. The benefit of the night>1 model’s ability to positively detect kills at sites (86% for night>1 model vs. 75% for full model) may have outweighed the cost of over attributing kills to clusters where none were found (40% for night>1 model vs. 25% for full model) (Table 4). Field crews often searched sites many days after the cluster had formed and it was likely that they failed to find evidence of some kills that actually occurred. Given the potential bias in assigning kills to non-kill clusters, it is reasonable to assume the model that over attributed kills to clusters was more reliable. 

**Table 4:** Individual cougar kill rates as determined with the >1 night model, and full model criteria, and sex and season.

Cat ID	Sex	Days monitored	Season	>1 night Model No. Kills	>1 Model Kill Rate (Kills/Day)	Full Model No. Kills	Full Model Kill Rate (Kills/Day)
F13	F	69	Winter	14	0.20	7	0.10
F13	F	86	Summer	16	0.19	15	0.17
F13	F	35	Winter	7	0.20	6	0.17
F13	F	119	Winter	22	0.18	16	0.13
F13	F	183	Summer	29	0.16	23	0.13
F13	F	149	Winter	17	0.11	15	0.10
F13	F	63	Winter	13	0.21	10	0.16
F13	F	169	Summer	23	0.14	20	0.12
F27	F	20	Winter	3	0.15	2	0.10
F27	F	25	Summer	3	0.12	3	0.12
F30	F	27	Summer	3	0.11	3	0.11
F47	F	36	Summer	5	0.14	4	0.11
F47	F	182	Winter	31	0.17	20	0.11
F47	F	183	Summer	26	0.14	22	0.12
F51	F	182	Winter	31	0.17	21	0.12
F51	F	183	Summer	30	0.16	21	0.11
F51	F	183	Winter	30	0.16	19	0.10
F51	F	112	Summer	13	0.12	10	0.09
F57	F	160	Winter	24	0.15	20	0.13
F57	F	183	Summer	24	0.13	20	0.11
F57	F	182	Winter	24	0.13	16	0.09
F57	F	183	Summer	20	0.11	15	0.08
F57	F	165	Winter	21	0.13	15	0.09
F61	F	91	Winter	14	0.15	9	0.10
F61	F	134	Summer	15	0.11	12	0.09
F69	F	26	Winter	3	0.12	3	0.12
F69	F	156	Winter	19	0.12	15	0.10
F69	F	183	Summer	18	0.10	19	0.10
F69	F	182	Winter	18	0.10	16	0.09
F69	F	183	Summer	20	0.11	22	0.12
F69	F	34	Winter	3	0.09	3	0.09
F101	F	91	Winter	8	0.09	8	0.09
F101	F	167	Summer	27	0.16	18	0.11
F101	F	58	Winter	9	0.16	9	0.16
F101	F	183	Summer	30	0.16	23	0.13
F101	F	72	Winter	11	0.15	10	0.14
F101	F	35	Summer	6	0.17	5	0.14
F101	F	183	Winter	22	0.12	19	0.10
F101	F	183	Summer	18	0.10	15	0.08
F101	F	175	Winter	20	0.11	16	0.09
F104	F	35	Summer	4	0.11	3	0.09
F109	F	27	Winter	5	0.19	4	0.15
F109	F	183	Summer	30	0.16	24	0.13
F109	F	183	Winter	29	0.16	22	0.12
F109	F	44	Summer	12	0.27	10	0.23
M21	M	183	Winter	21	0.11	20	0.11
M21	M	183	Summer	16	0.09	19	0.10
M21	M	182	Winter	16	0.09	16	0.09
M21	M	183	Summer	14	0.08	15	0.08
M21	M	182	Winter	15	0.08	17	0.09
M21	M	183	Summer	27	0.15	27	0.15
M21	M	182	Winter	27	0.15	24	0.13
M21	M	183	Summer	20	0.11	25	0.14
M21	M	114	Winter	17	0.15	15	0.13
M28	M	159	Winter	26	0.16	19	0.12
M28	M	177	Summer	24	0.14	18	0.10
M62	M	140	Winter	22	0.16	13	0.09
M62	M	183	Summer	23	0.13	19	0.10
M62	M	183	Winter	18	0.10	18	0.10
M62	M	77	Summer	6	0.08	7	0.09
M70	M	138	Winter	11	0.08	11	0.08
M70	M	183	Summer	14	0.08	12	0.07
M101	M	117	Winter	15	0.13	13	0.11
M101	M	75	Summer	12	0.16	14	0.19
M101	M	22	Winter	2	0.09	1	0.05
M101	M	69	Summer	11	0.16	12	0.17

Based on 211 ungulates of known age killed in summer and 382 ungulates of known age in winter, cougars as a population selected equal proportions of adult (37.4% in summer vs. 45.3% in winter, *z* = 1.88, P = 0.06) and subadult (14.7% in summer vs. 15.2% in winter, *z* = 0.163, P = 0.87) ungulates in summer and winter. Cougars did kill higher proportions of ungulates < 1 year of age in summer (47.9% in summer vs. 39.5% in winter, *z* = 1.97, P = 0.05), however, ungulate kill rates did not vary by season (F_1,52.32_=0.42, P=0.52) or between males and females (F_1,13.59_=0.11, P=0.52). Kills rates are reported in Table 4.

### Seasonal cougar ranges

We analyzed the seasonal ranges of 16 cougars. Of these, three home ranges were quantified from locations obtained from fixed-wing telemetry of VHF collars, seven from GPS locations, and the remaining from a combination of VHF and GPS locations. Mean overlap between summer and winter ranges was 59.8% ± 4.91 (range of 19.7 to 92%). 

**Figure 2:**
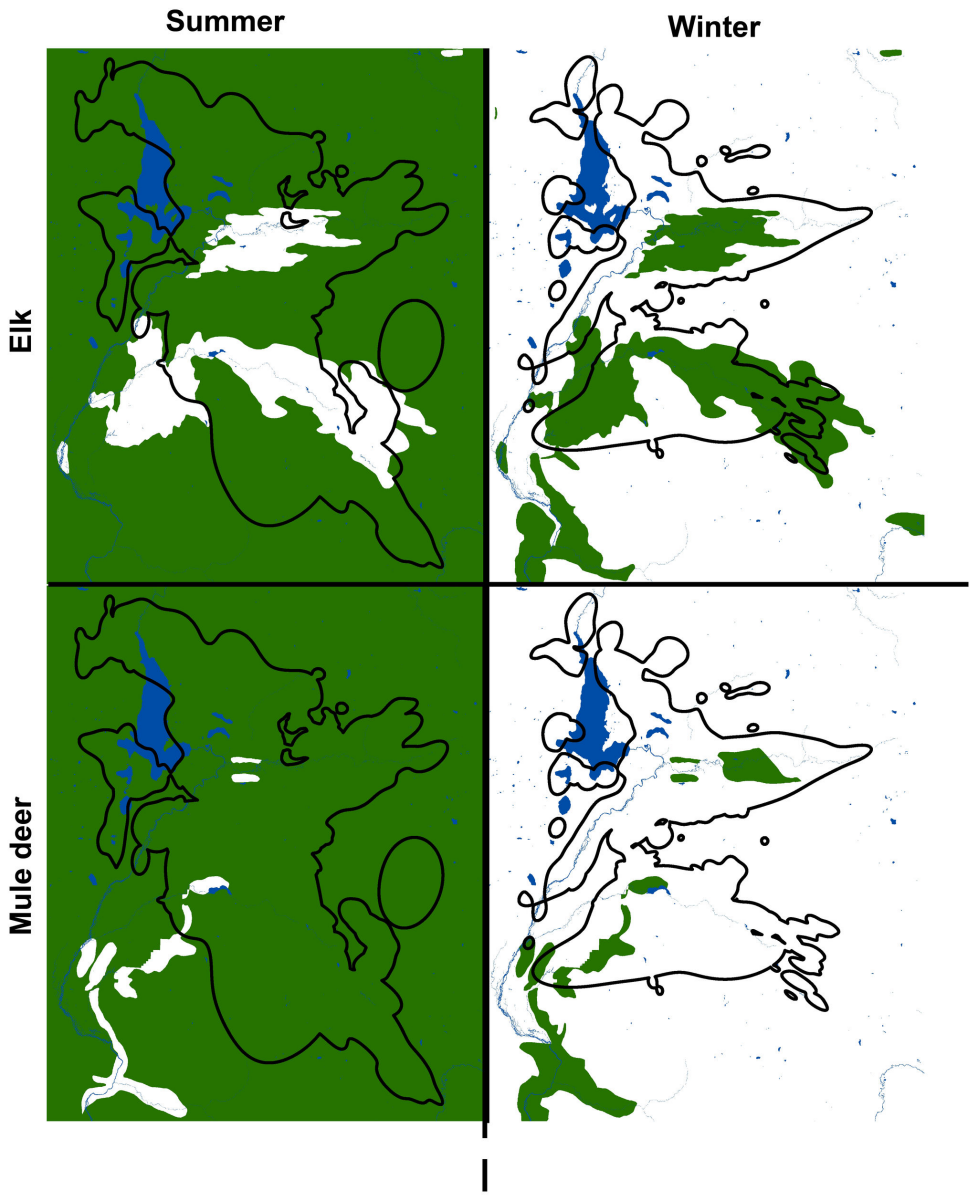
Seasonal ranges of mule deer, elk, and cougars in the study area. Seasonal ranges of mule deer and elk in green in the study area, as they overlap with the seasonal ranges of cougars outlined in black. The large lake is Jackson Lake in Grand Teton National Park.

**Figure 3:**
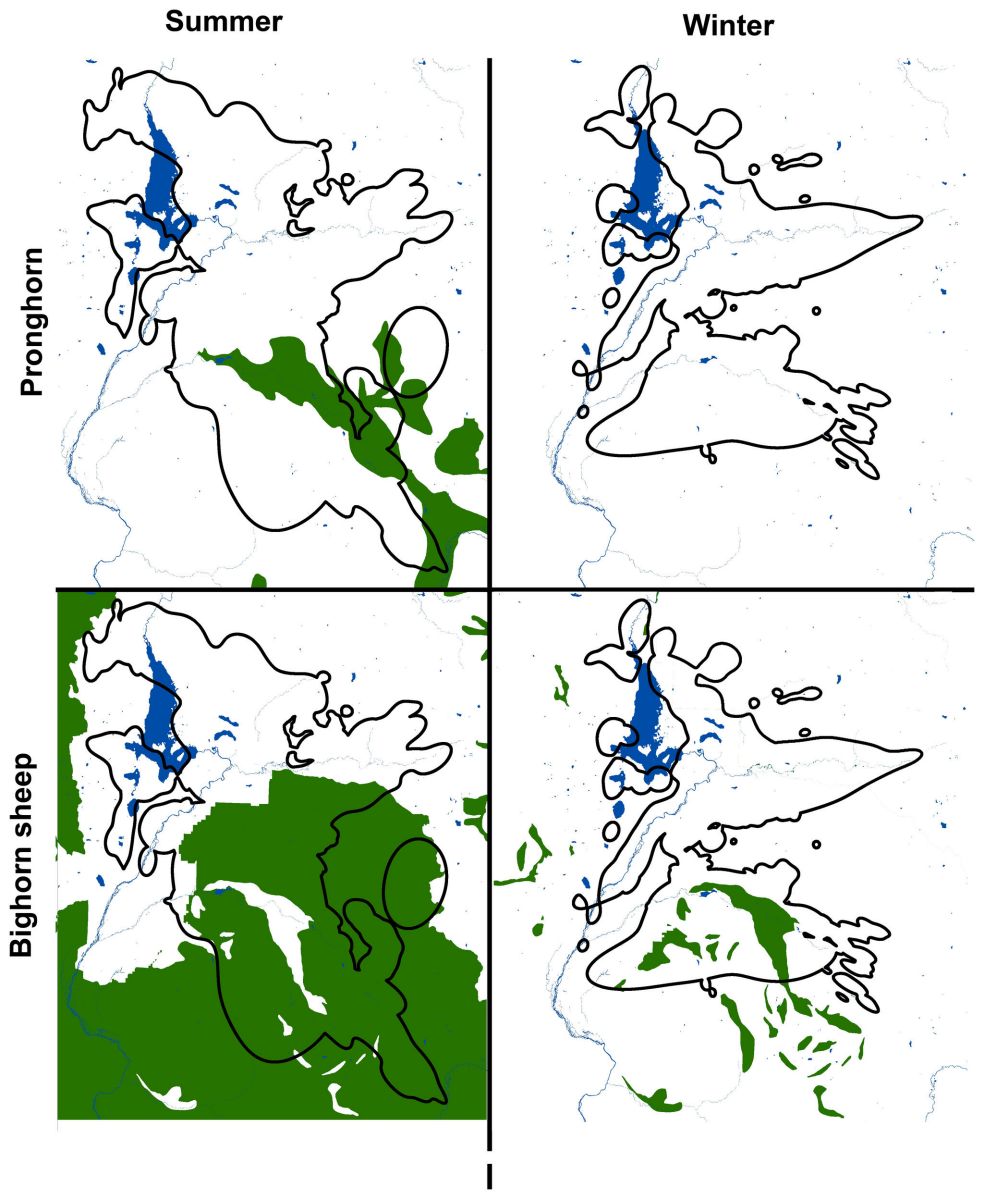
Seasonal ranges of pronghorn, bighorn sheep, and cougars in the study area. Seasonal ranges of pronghorn and bighorn sheep in green in the study area, as they overlap with the seasonal ranges of cougars outlined in black. The large lake is Jackson Lake in Grand Teton National Park.

The winter seasonal range of the marked cougar population overlapped 62% with the marked cougar summer range (Figures 2, 3). Forty-nine percent of cougar summer range overlapped with summer bighorn sheep range, 11% of cougar summer range overlapped with summer pronghorn range, 99% of cougar summer range overlapped with summer mule deer range, and 80% of cougar summer range overlapped with summer elk range (Figures 2, 3). Twenty-four percent of cougar winter range overlapped with winter bighorn sheep range, 0% of cougar winter range overlapped with winter pronghorn range, 7% of cougar winter range overlapped with winter mule deer range, and 36% of cougar winter range overlapped with winter elk range (Figures 2, 3).

### Seasonal hunting areas

In winter, the relative probability that a hunting cougar would kill prey at a particular place on the landscape increased as: 1) elevation decreased (β = -0.005115), 2) distance to edge habitat decreased (β = -0.00633), 3) distance to large bodies of water and rivers decreased (β = -0.00069), and 4) steepness increased (β = 0.09395). Relative to categorical variables, cougars in winter were less likely to make a kill on North- and East- facing slopes compared to South-facing. There was no statistical difference in the probability of a cougar killing on either South- or West- facing slopes. The probability of a cougar kill was lower in open habitats, such as meadows or pastures, compared to sagebrush-dominated communities, and the likeliness of a kill was similar among forested landscapes, riparian zones, and sagebrush communities (Table 5). 

**Table 5:** Odds ratios for top ranked resource selection function model of seasonal kill sites by cougars.

	Winter			Summer			
Parameter	Point estimate	Lower 95% CI	Upper 95% CI	Point estimate	Lower 95% CI	Upper 95% CI
Elevation	0.995	0.994	0.996	0.999	0.998	1.000
Edge	0.994	0.992	0.995	0.996	0.994	0.997
H2O	0.999	0.999	1.000	1.001	1.000	1.001
Slope	1.100	1.082	1.119			
North	0.622	0.412	0.939			
East	0.553	0.394	0.775			
West	0.757	0.566	1.013			
Meadow	0.473	0.287	0.779			
Barren	n/a	n/a	n/a			
Forested	0.909	0.694	1.191			
Riparian	0.764	0.450	1.298			

For categorical variables (aspect and habitat type) south aspects and sagebrush steppe were used as the reference category.

**Figure 4:**
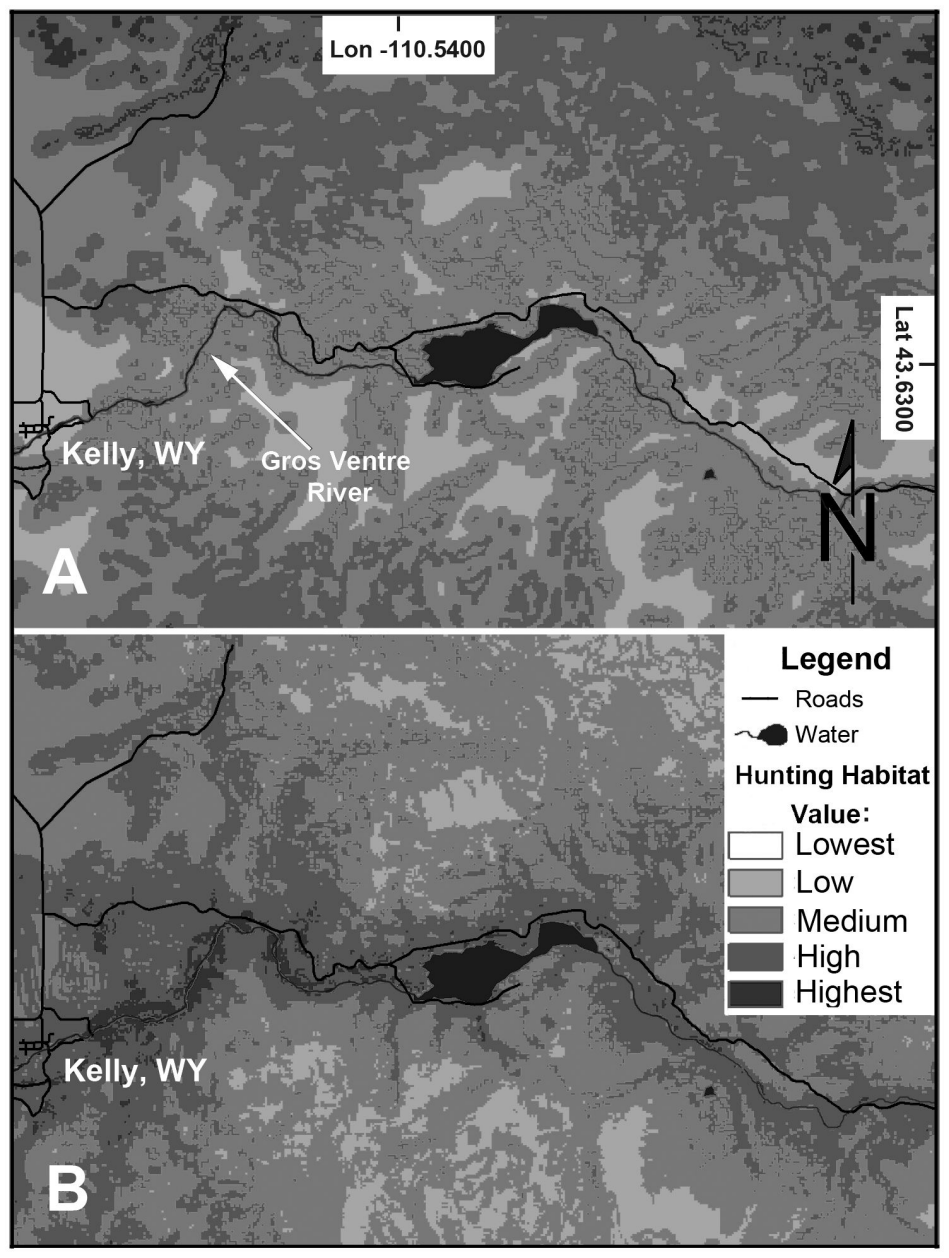
Seasonal habitat use by cougars. A comparison between Summer (A) and Winter (B) habitats utilized by cougars projected across the landscape based upon outputs from our Resource Selection Function outputs. The central body of water is Lower Slide Lake in the Bridger-Teton National Forest. Note the position of the Gros Ventre River, which we highlighted to emphasize the influence of “distance to water” in seasonal hunting.

During summer, cougars were more likely to make a kill in areas with: 1) decreasing elevation (β = -0.000865), 2) decreasing distance to edge habitat (β = -0.00495), and 3) increasing distance from large bodies of water and rivers (β = 0.000616; Table 5). Neither slope, aspect, nor vegetation type were significant in predicting kill site selection during summer. Terrain ruggedness was not a strong determinant in kill site selection during either season. Cross-validation analyses indicated that resource-selection functions were highly predictive for both winter and summer kill site selections (winter *r*_*s*_ = 0.97, *r* ^2^ = 0.94; summer *r*_*s*_ = 0.83, *r* ^2^ = 0.68). We used our model averaged parameter estimates to map the probability surface of cougars successfully killing prey across the landscape (Figure 4).

## Discussion

Whereas some cougars elsewhere follow migrating prey [4], they primarily remain sedentary in the Southern Yellowstone Ecosystem while their prey base change with the seasons. This is further evidence of the plasticity of this wide-ranging predator, and suggests that like wolves [7], cougars exhibit variable foraging strategies in systems with migrating prey. No doubt cougars in the SYE exhibited seasonal prey selection due to seasonal variation in prey vulnerability [8,10], but our data also supported the notion that cougar prey selection was also due to prey availability driven by seasonal variation in the availability of abundant, migratory elk and mule deer (Figure 3). We found support for our hypothesis that cougars would specialize on deer in summer and elk in winter, though only female cougars exhibited this pattern. Male cougars specialized on elk in both seasons, but increased their predation on deer in summer. 

Cougars also exhibited seasonal predation of several rare species. In a pattern very similar to that reported in Johnson et al. [2], bighorn sheep were almost exclusively killed in winter, when they dropped in elevation and congregated in larger groups in areas overlapping with abundant elk on winter feed grounds. In contrast, migratory pronghorn were killed almost exclusively in summer when overlapping with abundant mule deer on their summer range. In both cases, an abundant primary prey supported the cougar population, creating the potential for apparent competition scenarios that may be impacting rare prey. Nevertheless, we did not detect a single bighorn sheep or pronghorn killed by a male cougar, and only 9 of 38 (24%) cougars for which we documented prey selection killed moose. This suggests that bighorn sheep, pronghorn, and moose predation is likely stochastic and driven by the presence of individual cougars that select for these prey [22,48], as well as influenced by seasonal shifts in prey abundances and distributions. Only 25% of females and 0 males killed bighorn sheep; only 11% of females and 0 males killed pronghorn. Further, this suggests that any population-level management of cougars to protect rare ungulates in the SYE, such as increasing harvest quotas, would prove ineffective, unless by chance those individual cougars that select for these rare prey species were removed in the process [22]. 

We did not find support for our prediction that kill rates would be higher in summer than winter, as reported for southern Alberta by Knopff et al. [11]. Whereas cougars did select for younger animals in summer, they may abandon more meat in winter due to scavengers or ice, which can limit their access to carcasses in cold temperatures. However, our model outputs for kill rates were likely influenced by the difficulty in finding kills too long after the cougar had departed, or when the prey was small (including ungulates up to several months of age). This would influence both our comparison of selection for younger ungulates and the predictive power of our kill rate models. The multiplicity of GPS fix rates employed over the life of the project also likely reduced the effectiveness of many explanatory variables in identifying clusters with and without kills. 

Cougars also exhibited seasonal habitat selection for hunting (Figure 4), which we expect was partially driven by prey switching and the habitat preferences of their seasonal prey, as well as winter snows which restrict movements of predators and prey in the SYE [7,17,18]. As cougars kill prey in different habitats through the year, they distribute resources for numerous scavengers and decomposers [49], as well as macronutrients and microbial biomass (e.g., 50), that in turn, influence distributions of flora and pollinators on the landscape. 

## Conclusions

Our research highlighted the dynamic nature of the landscapes that are the Southern Yellowstone Ecosystem, and the behavioral plasticity of cougars, a charismatic keystone carnivore. As ungulates migrated across the landscape with the seasons, cougars remained relatively stationary and killed different prey. Thus predator-prey modeling based upon one season of sampling would be biased, as it would not account for changing cougar prey selection through the seasons. This work also highlights that seasonal prey selection exhibited by stationary carnivores in systems with migratory prey is not only driven by changing prey vulnerability, but also by changing prey abundances. Seasonal ungulate migrations, including those by elk and bison driven by supplemental winter feeding provided by humans, may be creating apparent competition scenarios resulting in higher predation rates on bighorn sheep in winter and pronghorn in summer. Nevertheless, cougar predation on bighorn sheep, pronghorn, and moose in the SYE also appears to be influenced by individual prey selection. Thus population-level management of cougars seeking to aid rare prey in the SYE will likely prove frustrating; this is not a conclusion specific to the SYE, but will likely be true in any multi-prey system in which cougars exhibit individual differences in prey selection [22]. As they hunt through the seasons, cougars distribute a wealth in carcasses for scavengers, decomposers and floral communities, in more diverse locations than they would if ungulates did not migrate. 

## References

[1] Giroux MA, Berteaux D, Lecomte N, Gauthier G, Szor G et al. (2012) Benefiting from a migratory prey: spatio-temporal patterns in allochthonous subsidization of an arctic predator. J Anim Ecol 81: 533-542. doi:10.1111/j.1365-2656.2011.01944.x. PubMed: 22268371. 22268371

[2] Johnson HE, Hebblewhite M, Stephenson TR, German DW, Pierce BM et al. (2013) Evaluating apparent competition in limiting the recovery of an endangered ungulate. Oecologia 171: 295-307. doi:10.1007/s00442-012-2397-6. PubMed: 22791131. 22791131

[3] Schaller GB (1972) The Serengeti Lion: a Study of Predator-Prey Relations. University of Chicago Press, Chicago.

[4] Pierce BM, Bleich VC, Wehausen JD, Bowyer RT (1999) Migratory patterns of mountain lions: implications for social regulation and conservation. J Mamm 80: 986-992. doi:10.2307/1383269.

[5] Ballard WB, Ayres LA, Krausman PR, Reed DJ, Fancy SG (1997) Ecology of wolves in relation to a migratory caribou herd in northwest Alaska. Wildlife Monogr 135: 3-47.

[6] Fryxell JM, Sinclair ARE (1988) Causes and consequences of migration by large herbivores. Trends Ecol Evol 3: 237-241. doi:10.1016/0169-5347(88)90166-8. PubMed: 21227239. 21227239

[7] Nelson AA, Kauffman MJ, Middleton AD, Jimenez M, McWhirter D et al. (2012) Elk migration patterns and human activity influence wolf habitat use in the Greater Yellowstone Ecosystem. Ecol Appl 22: 2293-2307. doi:10.1890/11-1829.1. 23387126

[8] Owen-Smith N (2008) Changing vulnerability to predation related to season and sex in an African ungulate assemblage. Oikos 117: 602–610. doi:10.1111/j.0030-1299.2008.16309.x.

[9] Sand H, Wabakken P, Zimmermann B, Johansson O, Pedersen HC et al. (2008) Summer kill rates and predation pattern in wolf—moose system: can we rely on winter estimates? Oecologia 156: 53–64. doi:10.1007/s00442-008-0969-2. PubMed: 18270746. 18270746

[10] Metz MC, Smith DW, Smith JA, Vucetich JA, Stahler DR et al. (2012) Seasonal patterns of predation for gray wolves in the multi-prey system of Yellowstone National Park. J Anim Ecol 81: 553–563. doi:10.1111/j.1365-2656.2011.01945.x. PubMed: 22260633. 22260633

[11] Knopff KH, Knopff AA, Kortello A, Boyce MS (2010) Cougar kill rate and prey composition in a multiprey system. J Wild Manage 74: 1435-1447. doi:10.2193/2009-314.

[12] Hornocker M, Negri S (2010) Cougar Ecology and Conservation. University of Chicago Press, Chicago.

[13] Cooley HS, Robinson HS, Wielgus RB, Lambert CS (2008) Cougar prey selection in a white-tailed deer and mule deer community. J Wildl Manage 72: 99-106. doi:10.2193/2007-060.

[14] Husseman JS, Murray DL, Power G, Mack C, Wenger CR et al. (2003) Assessing differential prey selection patterns between two sympatric large carnivores. Oikos 101: 591-601. doi:10.1034/j.1600-0706.2003.12230.x.

[15] Atwood TC, Gese EM, Kunkel KE (2007) Comparative patterns of predation by cougars and recolonizing wolves in Montana's Madison Range. J Wild Manage 71: 1098-1106. doi:10.2193/2006-102.

[16] Ruth TK, Murphy K (2010) Cougar–prey relationships. In: Hornocker M Negri S Cougar: ecology and conservation. Chicago: University of Chicago Press pp. 138-162.

[17] Sawyer H, Lindzey F, McWhirter D (2005) Mule deer and pronghorn migration in western Wyoming. Wild Soc B 33: 1266-1273. Available online at: doi:10.2193/0091-7648(2005)33[1266:MDAPMI]2.0.CO;2

[18] Smith BL (2007) Migratory behavior of hunted elk. Northwest Sci 81: 251-264. doi:10.3955/0029-344X-81.4.251.

[19] Bartnick TR, Van Deelen TR, Quigley HB, Craighead D (2013) Variation in cougar predation habits during wolf recovery in the southern Greater Yellowstone Ecosystem. Can J Zool 91: 82-93. doi:10.1139/cjz-2012-0147.

[20] Ballard WB, Lutz D, Keegan TW, Carpenter JH, deVos JC Jr (2001) Deer–predator relationships: a review of recent North American studies with emphasis on mule and black-tailed deer. Wildl Soc Bull 29: 99-115.

[21] Eberhardt LL, White PJ, Garrott RA, Houston DB (2007) A seventy-year history of trends in Yellowstone's northern elk herd. J Wild Manage 71: 594-602. doi:10.2193/2005-770.

[22] Elbroch LM, Wittmer HU (2013) The effects of puma prey selection and specialization on less abundant prey in Patagonia. J Mamm 94: 259-268. doi:10.1644/12-MAMM-A-041.1.

[23] Quigley K (2000) Immobilization and biological sampling protocols. Hornocker Wildlife Institute/Wildlife Conservation Society, Moscow.

[24] Sikes RS, Gannon WL, the Animal Care and Use Committee of the American Society of Mammalogists (2011) Guidelines of the American Society of Mammalogists for the use of wild mammals in research. J Mamm 92: 235-253. doi:10.1644/10-MAMM-F-355.1. PMC5909806 29692469

[25] Knight DH (1996) Mountains and plains: the ecology of Wyoming landscapes. Yale University Press, New Haven.

[26] Anderson CR, Lindzey FG (2003) Estimating cougar predation rates from GPS location clusters. J Wild Manage 67: 307-316. doi:10.2307/3802772.

[27] Heffelfinger J (2010) Age criteria for Southwestern game animals Special Report #19 Arizona Game and Fish Department, Arizona.

[28] Knopff KH, Knopff AA, Warren MB, Boyce MS (2009) Evaluating global positioning system telemetry techniques for estimating cougar predation parameters. J Wild Manage 73: 586-597. doi:10.2193/2008-294.

[29] Miller CS, Hebblewhite M, Petrunenko YK, Seryodkin IV, DeCesare NJ, et al. (2013) Estimating Amur tiger (*Panthera* *tigris* *altaica*) kill rates and potential consumption rates using global positioning system collars. J Mamm. In Press

[30] Ruth TK, Buotte PC, Quigley HB (2010) Comparing ground telemetry and Global Positioning System methods to determine cougar kill rates. J Wild Manage 74: 1122-1133. doi:10.2193/2009-058.

[31] Hosmer DW, Lemshow S (2000) Applied Logistic Regression. John Wiley and Sons, New York.

[32] Manly B, McDonald L, Thomas D (1993) Resource Selection by Animals. Chapman & Hall, New York.

[33] Worton BJ (1989) Kernel methods for estimating the utilization distribution in home-range studies. Ecology 70: 164-168. doi:10.2307/1938423.

[34] Kie JG, Matthiopoulos J, Fieberg J, Powell RA, Cagnacci F et al. (2010) The home-range concept: are traditional estimators still relevant with modern telemetry technology? Philos T R Soc B 365: 2221-2231. doi:10.1098/rstb.2010.0093. PubMed: 20566499. PMC2894967 20566499

[35] Beyer HL (2009–2012) Geospatial Modeling Environment. Available: http://www.spatialecology.com/gme/ /. Accessed 2012 May 3

[36] Loader CR (1999) Bandwidth selection: classical or plug-in? Ann Stat 27: 415-438. doi:10.1214/aos/1018031200.

[37] Sappington JM, Longshore KM, Thompson DB (2007) Quantifying landscape ruggedness for animal habitat analysis: a case study using bighorn sheep in the Mojave Desert. J Wild Manage 71: 1419-1426. doi:10.2193/2005-723.

[38] Stewart KM, Bowyer RT, Kie JG, Hurley MA (2010) Spatial distributions of mule deer and North American elk: resource partitioning in a sage-steppe environment. Am Midl Nat 163: 400-412. doi:10.1674/0003-0031-163.2.400.

[39] SAS Institute Inc. (1990) SAS/STAT user’s guide, 6.03. SAS Institute Inc, Cary, NC.

[40] Compton BW, Rhymer JM, McCollough M (2002) Habitat selection by wood turtles: an application of paired logistic regression. Ecology 83: 833-843. Available online at: doi:10.1890/0012-9658(2002)083[0833:HSBWTC]2.0.CO;2

[41] Boyce MS (2006) Scale for resource selection functions. Divers Distrib 12: 269-276. doi:10.1111/j.1366-9516.2006.00243.x.

[42] Long RA, Kie JG, Bowyer RT, Hurley MA (2009) Resource selection and movements by female mule deer: effects of reproductive stage. Wild Biol 15: 288-298.

[43] Lendrum PE, Anderson CR, Long RA, Kie JG, Bowyer RT (2012) Habitat selection by mule deer during migration: effects of landscape structure and natural-gas development. Ecosphere 3: art82

[44] Burnham KP, Anderson DR (2002) Model selection and multimodel inference: a practical information theoretic approach, 2nd ed. Springer-Verlag.

[45] Boyce MS, Mao JS, Merrill EH, Fortin D, Turner MG et al. (2003) Scale and heterogeneity in habitat selection by elk in Yellowstone National Park. Ecoscience 10: 421-431.

[46] Kauffman MJ, Varley N, Smith DW, Stahler DR, MacNulty DR et al. (2007) Landscape heterogeneity shapes predation in a newly restored predator-prey system. Ecol Lett 10: 690-700. doi:10.1111/j.1461-0248.2007.01059.x. PubMed: 17594424. 17594424

[47] Kunkel KE, Ruth TK, Atwood TC, Pletscher DH, Hornocker MG (2013) Assessing the value of wolves and cougars as conservation surrogates by linking carnivore hunting success with landscape characteristics. Anim Conserv 16: 32-40. doi:10.1111/j.1469-1795.2012.00568.x.

[48] Festa-Bianchet M, Coulson T, Gaillard JM, Hogg JT, Pelletier F (2006) Stochastic predation events and population persistence in bighorn sheep. Proc Biol Sci 273: 1537-1543. PubMed: 16777749. 16777749 10.1098/rspb.2006.3467 PMC1560313

[49] Elbroch LM, Wittmer HU (2013) Nuisance ecology: do scavenging condors exact foraging costs on pumas in Patagonia? PLOS ONE 8: e53595. doi:10.1371/journal.pone.0053595. PubMed: 23301093. 23301093 PMC3536754

[50] Bump JK, Peterson RO, Vucetich JA (2009) Wolves modulate soil nutrient heterogeneity and foliar nitrogen by configuring the distribution of ungulate carcasses. Ecology 90: 3159-3167. doi:10.1890/09-0292.1. PubMed: 19967871. 19967871

